# Environmental Life Cycle Assessment of Diets with Improved Omega-3 Fatty Acid Profiles

**DOI:** 10.1371/journal.pone.0160397

**Published:** 2016-08-09

**Authors:** Carla R. V. Coelho, Franck Pernollet, Hayo M. G. van der Werf

**Affiliations:** SAS, Agrocampus Ouest, INRA, Rennes, France; INIA, SPAIN

## Abstract

A high incidence of cardiovascular disease is observed worldwide, and dietary habits are one of the risk factors for these diseases. Omega-3 polyunsaturated fatty acids in the diet help to prevent cardiovascular disease. We used life cycle assessment to analyse the potential of two strategies to improve the nutritional and environmental characteristics of French diets: 1) modifying diets by changing the quantities and proportions of foods and 2) increasing the omega-3 contents in diets by replacing mainly animal foods with equivalent animal foods having higher omega-3 contents. We also investigated other possibilities for reducing environmental impacts. Our results showed that a diet compliant with nutritional recommendations for macronutrients had fewer environmental impacts than the current average French diet. Moving from an omnivorous to a vegetarian diet further reduced environmental impacts. Increasing the omega-3 contents in animal rations increased Eicosapentaenoic Acid (EPA) and Docosahexaenoic Acid (DHA) in animal food products. Providing these enriched animal foods in human diets increased their EPA and DHA contents without affecting their environmental impacts. However, in diets that did not contain fish, EPA and DHA contents were well below the levels recommended by health authorities, despite the inclusion of animal products enriched in EPA and DHA. Reducing meat consumption and avoidable waste at home are two main avenues for reducing environmental impacts of diets.

## Introduction

The world faces an epidemic of preventable illnesses such as cardiovascular diseases (CVD). According to estimates from the World Health Organization [1], 30% of deaths worldwide are caused by CVD, and in Europe it causes 42% of all deaths in men [2]. According to Calder [3] and Mozaffarian and Rimm [4], the intake of omega-3 polyunsaturated fatty acids (hereafter referred to as “omega-3”) has been negatively correlated with the occurrence of CVD. It is also noteworthy that lower incidence of CVD has been associated with vegetarian diets [5–8]. Several studies, mostly focusing on greenhouse gas emissions, have consistently shown that, compared to average current diets, diets compliant with nutritional recommendations and vegetarian diets have lower environmental impacts [9–11].

The European Food Safety Authority [12] recommends an intake of 250 mg/day of omega-3 Eicosapentaenoic Acid (EPA) and Docosahexaenoic Acid (DHA); the French recommendation is 500mg/day of EPA and DHA [13], and the International Society for the Study of Fatty Acids and Lipids [14] recommends an intake of 500 mg/day of EPA and DHA for adults for cardiovascular health. While the main sources of EPA and DHA are fish and seafood [15], 80% of fish stocks worldwide are considered fully exploited, overexploited or depleted, meaning there is very little room to increase the harvest [16]. This conflict between health recommendations and preservation of fish stocks was highlighted by Macdiarmid [17] and was also investigated in terms of consumer choices by Clonan, et al. [18]. Motivated by improving the nutritional quality of food products, research has shown that increasing the omega-3 content in animal feed rations can result in higher omega-3 contents in animal products [19]. This practice can contribute to improving omega-3 levels in human diets without increasing pressure on fish stocks.

This paper builds on Pernollet, et al. [20] and estimates environmental impacts and levels of omega-3 fatty acid intake in diets corresponding to four food consumption patterns and two levels of omega-3. The objective is to answer three main questions: 1) What are the environmental impacts of the current French diet and omnivorous and vegetarian diets compliant with French nutritional guidelines at the macronutrient level? 2) Does improving the omega-3 profile of diets affect their environmental impacts? 3) Which improvement options can reduce environmental impacts of diets?

## Methodology

### Diet design

**Fig 1:**
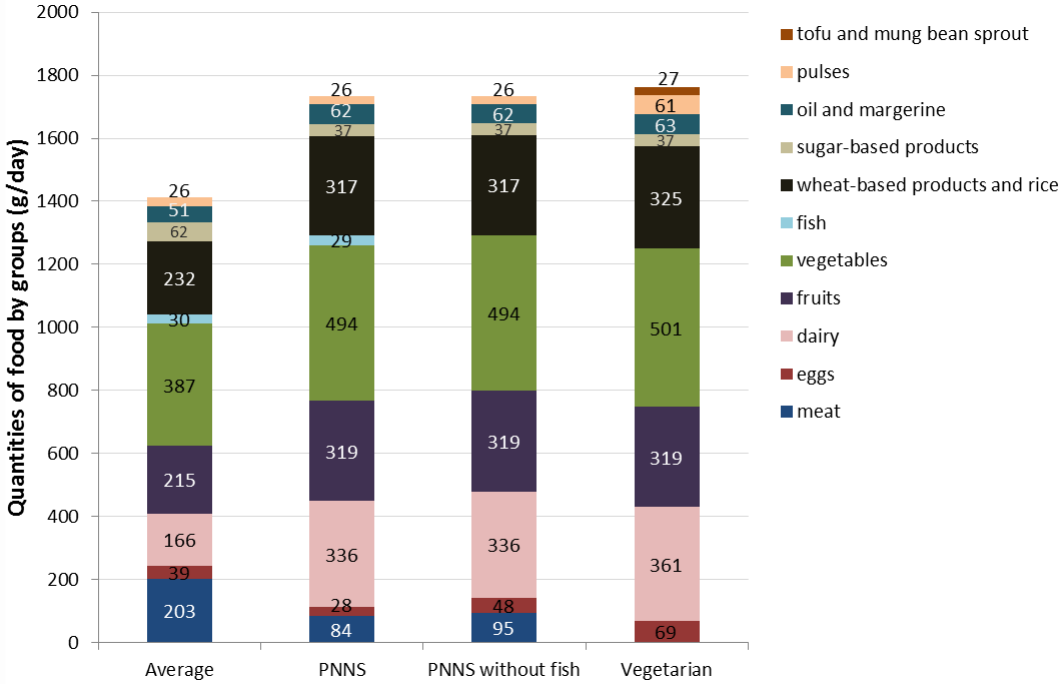
Composition of the four diets, in grams of food per day by food group.

The study consisted of assessing four diets which were based, as closely as possible, on French habits of food consumption. An average diet for a 15-day period (hereafter referred to as Average diet) was designed, consisting of 105 foods representing breakfast, lunch, and dinner, excluding alcohol. The Average diet was adapted from survey data from 2010 on Nutritional Behaviour and Food Consumption in France (*Comportement et Consommation Alimentaire en France*) to approximate food consumption of an adult French man [21]. The Average diet was modified to obtain three diets complying with nutritional recommendations for macronutrients from the French National Programme for Nutrition and Health (*Programme National Nutrition Santé*, PNNS)[22]: 1) PNNS, a diet consisting of the same foods as Average, but in different amounts; 2) PNNS without fish, similar to PNNS, but without fish; and 3) Vegetarian, similar to PNNS but without fish and meat. Compared to the Average diet, the daily intake of the other three diets was mainly modified as follows: a reduction of at least 108 g of meat and 25 g of sugar and an increase of 170–195 g of dairy, 104 g of fruit, 107–114 g of vegetables, and 85–93 g of wheat and rice (Fig 1). In the 15-day period, compared to the Average diet, the PNNS diet had 15 g less of hake and cod each, 20 g less of tuna and 45 g more of salmon. The diets contained some dishes prepared with several ingredients (e.g. pizza, spaghetti bolognese, shepherd’s pie), these are hereafter referred to as homemade dishes. A detailed description of foods items and diets is given in the Supporting information (S1 Table).

A version of each of the four diets with increased omega-3 contents was created. This version was called Bleu-Blanc-Coeur (BBC), after the name of a French association which promotes changes to animal feed to provide food products with higher omega-3 contents. In BBC versions of the diets, all meat, egg and dairy food products were replaced with their BBC versions. Also, 2.5% of the flour used in bread and in homemade dishes was replaced with linseed flour; for pasta, the flour was standard, but the eggs were BBC. In BBC versions of diets, margarine contained less sunflower oil and palm oil and more rapeseed oil. In homemade dishes and salad dressing, sunflower oil was replaced with a mix of 28% rapeseed oil and 72% olive oil. Hereafter, food items and diets with higher omega-3 contents are described as “BBC version” or simply “BBC”.

### Omega-3 contents

BBC versions of animal products come from animals fed modified rations (more alfalfa, rapeseed meal, sunflower meal and linseed; less soya bean meal, maize, barley and wheat). Depending on the type of animal, these modifications in animal rations can also result in certain improvements in animal production, such decreased enteric CH_4_ emissions, increased average daily weight gain of animals and increased survival rate of piglets. Details on the changes in ration per animal type and the animal-production improvements considered in this study are presented in S2 Table. For the human diets, nutritional profiles of the food items were obtained from the French food composition table [23], while EPA and DHA contents of both standard and BBC versions of animal foods were provided by the *Bleu-Blanc-Coeur* association (Mathieu Guillevic, pers. comm., 2015) (S3 Table).

### Life cycle assessment

Life cycle assessment (LCA) is a standardised framework for systematic evaluation of environmental aspects of a product or service system through all stages of its life cycle [24]. It consists of assessing environmental impacts of a product from extraction of raw materials to product disposal [25].

Environmental impacts in LCA are presented relative to a functional unit. In this study the unit was “average daily kcal-adjusted food ingestion for one person in a French two-person urban household”. Although designed to be isocaloric, the diets used realistic quantities of food items such as ‘one egg’; as a consequence, they contained from 2174–2314 kcal/person/day. To be consistent with LCA methodology, all diets were adjusted to 2300 kcal/person/day. For each diet, results were calculated according to the full cradle to mouth method described by Pernollet, et al. [20], and represent impacts associated with production, transformation, transport, distribution, storage, cooking (when applicable), losses throughout the supply chain and waste at home. Waste at home was classified and accounted for according to Quested, et al. [26], who defined three waste types: avoidable, potentially avoidable and non-avoidable.

In the Life Cycle Inventory Analysis (LCI) phase, inputs from the environment (resources used) and outputs to the environment (emissions) associated with the product are listed. We used inventory data from the AGRIBALYSE database version 1.1 [27] and the ecoinvent database version 2.2. Impacts were modelled using SimaPro 8.

In the Life Cycle Impact Assessment (LCIA) phase, inputs and outputs collected in the LCI phase are converted in impacts. This is done by multiplying the aggregated resources used and the aggregated emissions of each individual substance with a characterisation factor for each impact category to which it may potentially contribute [28]. Characterisation factors are substance-specific, quantitative representations of the additional environmental pressure per unit emission of a substance [29]. In this study the following environmental impact categories were assessed: global warming potential (GWP) using GWP100a; GWP including land use change (GWP-LUC) according to Audsley, et al. [30]; acidification (AC) and eutrophication (EU) using the CML IA baseline, April 2013; land occupation (LO) using the CML IA non- baseline, October 2012; Total Cumulative Energy Demand (CED) according to CED v1.08 from ecoinvent (renewable and non-renewable energy, excluding gross calorific energy in biomass); biotic natural resource-depletion species (BNR-spe) and biotic natural resource-depletion ecosystems (BNR-eco) [31]. The complete list of characterization factors is compiled in the datasheet presented in supplementary information (S1 Datasheet).

## Results

### Environmental impacts of animals and animal products at the farm gate

At the farm gate, BBC products had slightly lower GWP, GWP-LUC and CED impacts than standard products for cow milk, cattle, broilers, eggs and pigs, while these impacts were somewhat higher for goat milk and rabbits (Table 1). BBC products had slightly higher (cow milk, sheep milk, goat milk, rabbits) or lower (broilers, eggs) EU impact than standard products. Compared to standard products, BBC products had slightly higher LO for all products except cattle.

**Table 1:** Environmental impacts at the farm gate of products with higher omega-3 content (BBC) relative to those of standard products, in percent. A negative value indicates a reduction in the impact of the BBC product compared to the standard product; a positive value indicates an increase. Results for global warming potential (GWP), GWP including land use change (GWP-LUC), cumulative energy demand (CED), acidification (AC), eutrophication (EU), land occupation (LO).

	Percentage difference of impacts of BBC products compared to those of standard products					
Product	GWP	GWP-LUC	CED	AC	EU	LO
Cow milk	-3.4	-1.8	-8.5	-1.4	1.7	5.6
Sheep milk	-0.3	0.1	1.7	0.1	1.4	0.9
Goat milk	1.4	2.7	2.7	0.4	5.9	7.5
Cattle	-1.4	-1.2	-1.6	-1.1	-0.4	-0.3
Rabbits	1.6	2.5	2.1	0.8	6.8	7.4
Broilers	-2.8	-1.3	-11.1	-1.6	-2.1	4.6
Eggs	-5.5	-3.5	-12.9	-2.5	-2.5	2.5
Pigs	-1.5	-0.7	-2.5	-2.2	0.6	3.2

### Ingestion of EPA and DHA

**Fig 2:**
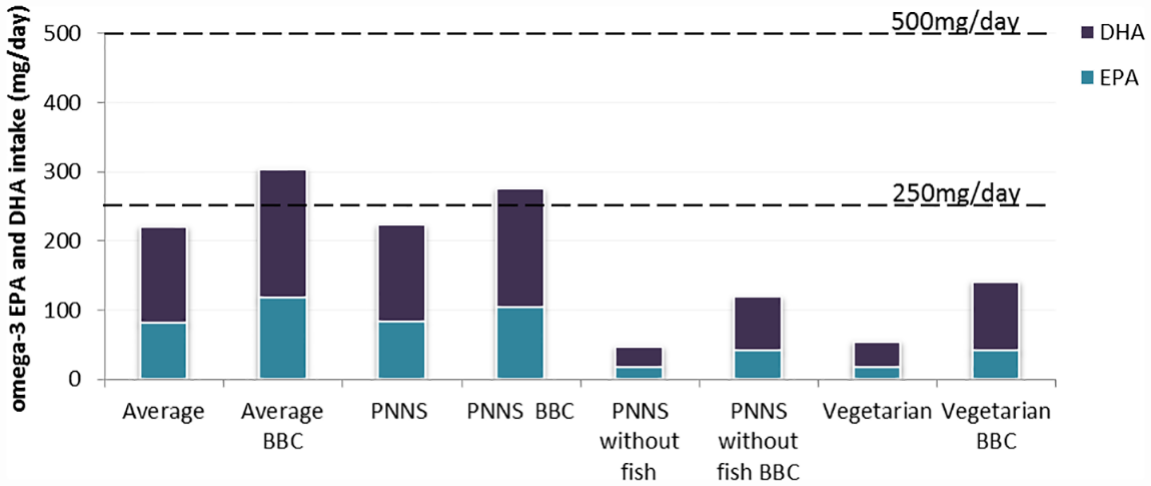
Estimated EPA and DHA ingestion for standard and BBC versions of the four diets. Horizontal lines represent recommended daily intake levels according to the European Food Safety Authority (250 mg/day) and according to ANSES, the French Agency for Food, Environmental and Occupational Health & Safety (500 mg/day).

For standard versions of the Average and PNNS diets, EPA and DHA ingestion was estimated to be approximately 220 mg/day, while for the PNNS without fish and Vegetarian diets, it was around 50 mg/day (Fig 2). Compared to standard versions, EPA and DHA ingestion for BBC versions increased by 52 (PNNS) to 87 (Vegetarian) g/day.

### Environmental impacts of diets

**Fig 3:**
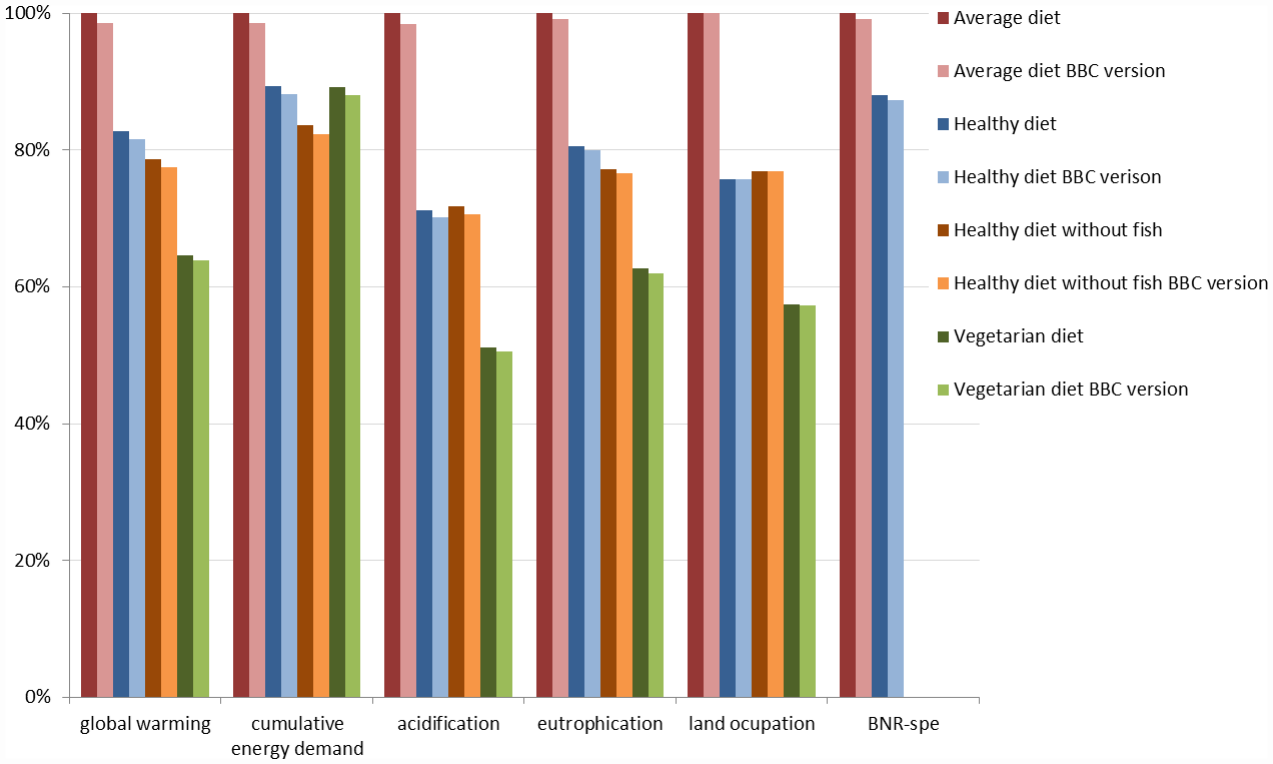
Environmental impact assessment results for global warming potential (GWP), cumulative energy demand (CED), acidification (AC), eutrophication (EU), land occupation (LO) and biotic natural resource-depletion species (BNR-spe). Results are relative to those of the Average diet.

Impacts of BBC versions of the diets were slightly lower than or similar to those of standard versions; differences did not exceed 1.5 percentage points (Fig 3). GWP and GWP-LUC had similar relative differences among diets. Compared to the Average diet, impacts of the PNNS diet were about 12% lower for CED and BNR-spe, 20% lower for GWP and EU, 24% lower for LO and 29% lower for AC. Differences between PNNS and PNNS without fish did not exceed 6 percentage points, except for BNR-spe, which was zero for PNNS without fish. Compared to the Average diet, impacts of the Vegetarian diet were lower: 11% for CED, 35% for GWP, 37% for EU, 43% for LO, 49% for AC, and 100% for BNR-spe. We limit presentation of BNR-eco to absolute values in the Supporting information, where absolute values for all impacts are presented (S4 Table). For the results of the life cycle impact assessment for all food items, consult supporting information (S5 Table).

**Fig 4:**
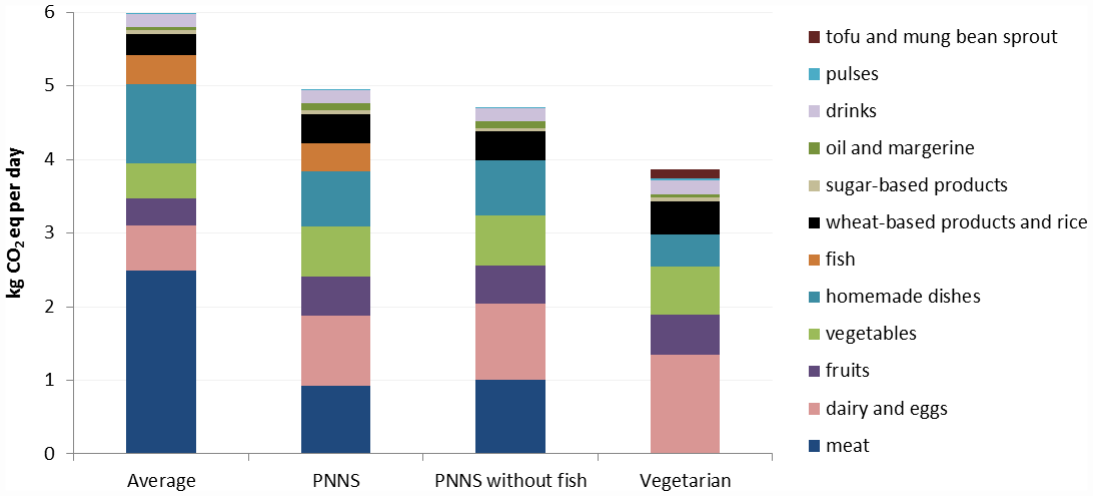
Contribution of food groups to the daily global warming potential impact of one person for the four diets.

**Fig 5:**
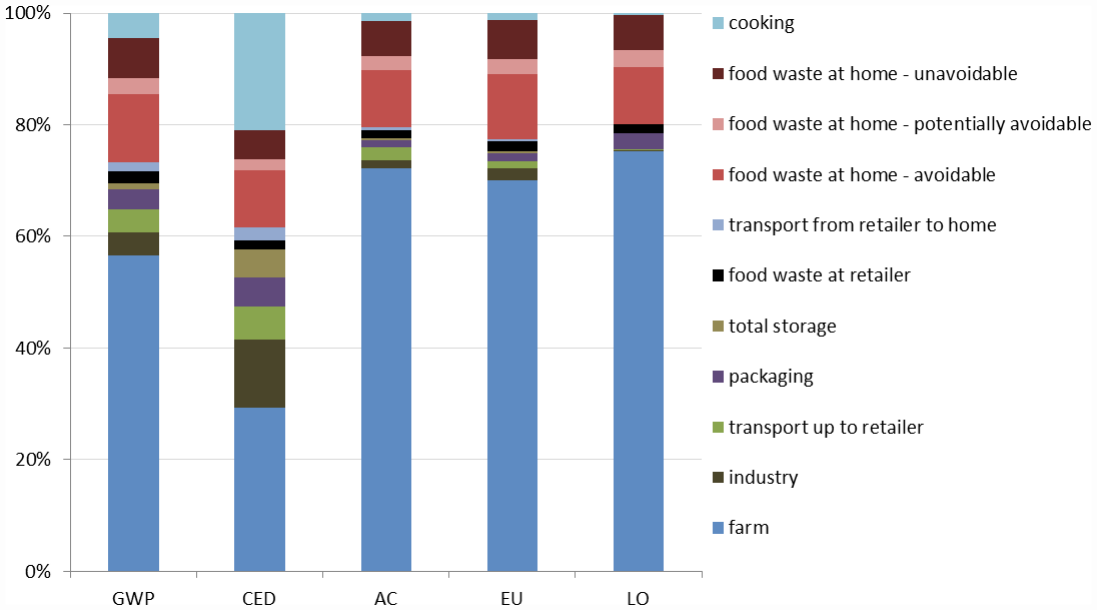
Contribution of life cycle stages of the Average diet to global warming potential (GWP), cumulative energy demand (CED), acidification (AC), eutrophication (EU), and land occupation (LO) impacts.

To identify improvement options for the diets, we assessed the contribution of food groups to GWP for the four diets (Fig 4) and the contribution of life-cycle stages to GWP, CED, AC, EU and LO for the Average diet (Fig 5). For the Average diet, meat contributed 42% to GWP, homemade dishes 18% and dairy and eggs 10% (Fig 4). The PNNS and PNNS without fish diets had similar contribution profiles, with meat, dairy and eggs together accounting for 38% and 44% of GWP for PNNS and PNNS without fish, respectively. For the Vegetarian diet, dairy and eggs contributed 37% of GWP and fruit and vegetables contributed 18%.

For CED, the farm stage (29%) and cooking (21%) contributed most. For the other impacts, farm stage contributed 57% to GWP, 70% to EU, 72% to AC and 75% to LO (Fig 5). Potentially avoidable and avoidable waste contributed 12–15%, and avoidable waste alone contributed at least 10%, of total impact in each category.

**Fig 6:**
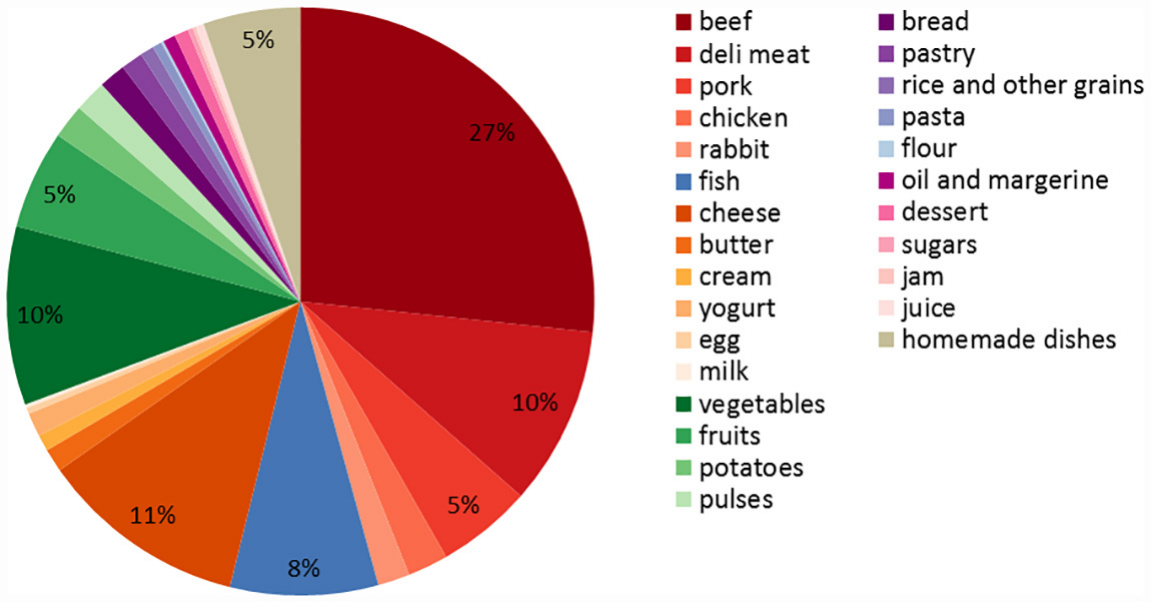
Contribution of food groups to global warming potential due to avoidable waste for the Average diet.

For the average diet, beef, cheese and deli meat together accounted for 48% of GWP of avoidable waste at home, while fruit and vegetables accounted for 16% (Fig 6). When analysed per food group, meat accounted for 42% of GWP, and dairy accounted for 10% for the average diet. The meat group does not include meat in homemade dishes (e.g. pizza, shepherd’s pie), which is a separate group.

### Other scenarios

**Fig 7:**
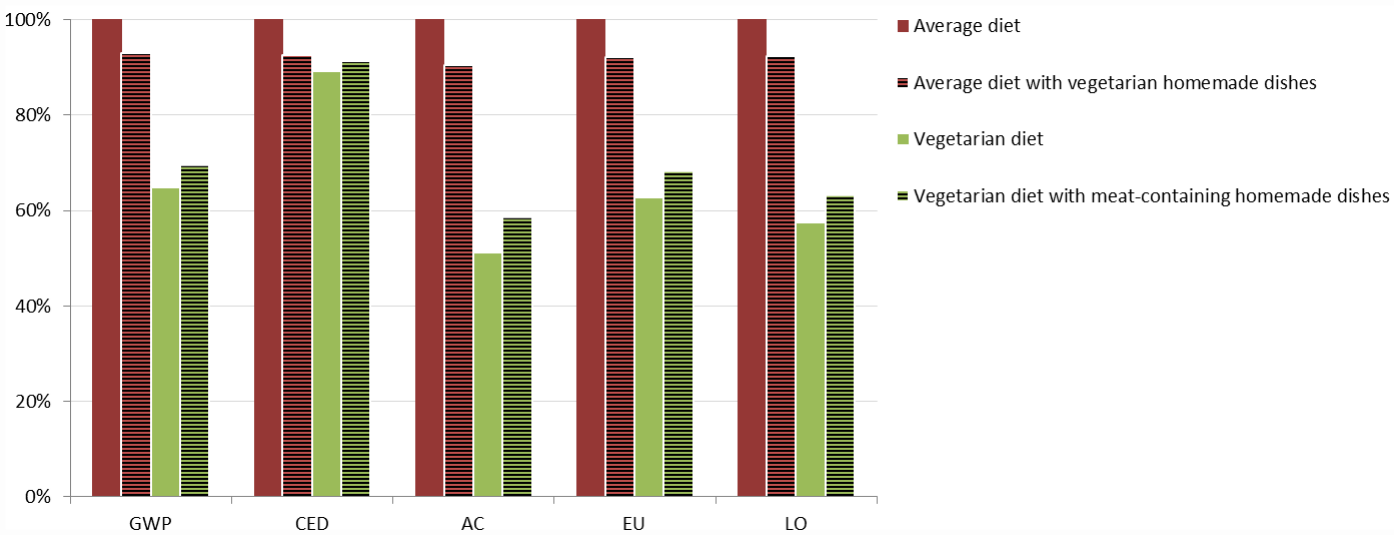
Impact values for global warming potential (GWP), cumulative energy demand (CED), acidification (AC), eutrophication (EU) and land occupation (LO) for the Average diet, Average diet with vegetarian homemade dishes, Vegetarian diet and Vegetarian diet with meat-containing homemade dishes. Results are relative to the average diet.

Keeping in mind that reducing meat consumption from around 200 g a day (as in the Average diet) to 0 g per day as in a vegetarian diet might be perceived as a radical change, we investigated the environmental impacts of two intermediate diet options: 1) replacing homemade dishes in the Average diet with vegetarian dishes and 2) including homemade dishes containing meat in the Vegetarian diet. The former decreased relative impacts by 7–9 percentage points, while the latter increased relative impacts by 2–8 percentage points (Fig 7).

## Discussion

### Impacts at farm gate of terrestrial animal products with higher omega-3 contents

Modifying animal rations to improve nutritional and environmental profiles of animal products has become an area of research interest [32,33]. We investigated the influence of such changes on potential environmental impacts of animal-production systems. At the farm gate, changes to animal rations aiming to increase omega-3 contents resulted in modest reductions in GWP and CED impacts for pig and cow. Reductions were somewhat larger for cow milk, egg and broiler, whereas slight increases in these impacts were observed for rabbit and goat milk. These same changes to animal rations led to increased LO for most animals and products. For sheep milk, goat milk and rabbit, increasing omega-3 content in these animals’ rations resulted in higher impacts in all categories, as quantities of soya bean meal in their diets did not decrease (unlike in the other animals’ diets) and no animal-production improvements occurred.

### Modification of human diets

Results of comparing environmental impacts of the Average and PNNS diets agree with literature findings, demonstrating that diets complying with nutritional guidelines or described as healthy have lower impacts than average current diets [10,11,34,35]. Thus, moving from the current diet to one which corresponds to nutritional recommendations is a win-win solution, as it simultaneously improves nutritional quality and reduces environmental impacts. Moving from the PNNS to the Vegetarian diet further reduced environmental impacts, confirming literature results [9,11]. While PNNS and Vegetarian diets both correspond to nutritional recommendations for macronutrients, EPA and DHA contents in Vegetarian diets were lower than in PNNS diets and were well below levels recommended by international and national health authorities. Therefore, the Vegetarian diet represents a challenge because, among the diets compared, it had both the lowest environmental impacts and the lowest supply of EPA and DHA. The low intake of EPA and DHA by vegetarians has been recognised in the literature [5] and, although evidence is lacking that vegetarians would derive additional cardiovascular benefit from increased EPA and DHA intake, increasing intake of EPA and DHA is not considered an unreasonable goal for vegetarians [36].

### Increasing omega-3 levels in human diets

Increasing the omega-3 content in human diets by replacing terrestrial animal foods, oils and flour with similar foods higher in omega-3 did not affect LO and slightly reduced GWP, CED, AC and EU in the diets studied, while increasing EPA and DHA intake by 52–87 mg/day without increasing the pressure on fish stocks. Despite improvement in the diets’ omega-3 profiles, none of them achieved the 500 mg/day recommended intake level of EPA and DHA. Adarme-Vega, et al. [37] suggested that oils from microalgae rich in EPA and DHA can be used to further supplement diets.

### Options to further reduce environmental impacts

In agreement with literature results, we identified reduction of waste at home as an area of potential reduction in impacts [38]. Interestingly, although meats and other animal foods have lower avoidable waste rates than other foods such as fruits and vegetables [26], our results showed that animal foods dominated environmental impacts of waste in an Average diet.

A mostly vegetarian diet, i.e. the Vegetarian diet with consumption of meat-containing homemade dishes, had the second-lowest impact in most categories. This indicates that this type of intermediate, less-radical diet merits consideration, as it may lead to wider adoption by average diet eaters and facilitate evolution towards more sustainable diets.

### Study limitations

The environmental impacts studied were limited to the five most used in LCA, with the addition of impacts of land-use change to the GWP category and assessment of pressure on fish stocks and marine ecosystems. Although desirable, assessing spatially differentiated impacts such as water stress or biodiversity loss due to land use was not possible due to lack of spatially differentiated inventory data and time constraints.

## Conclusions

We analysed the potential of two strategies for improving nutritional and environmental characteristics of French diets: 1) modifying diets by changing the quantities and proportions of foods and 2) increasing omega-3 contents in diets by replacing certain foods with equivalent foods with higher omega-3 contents while maintaining the same quantities and proportions of foods. We assessed environmental impacts of these strategies and investigated options which could further reduce environmental impacts from an LCA perceptive.

The Vegetarian diet had lower environmental impacts than the PNNS diet, which had lower environmental impacts than the current Average diet. Increasing omega-3 contents of the diets by including foods higher in omega-3 did not influence the diets’ environmental impacts. Such substitutions, however, can provide healthier diets without increasing environmental impacts or pressure on fish stocks. Animal foods dominated the environmental impacts of waste in the average current diet.

## Supporting Information

S1 Datasheet Compilation of impact assessment characterization factors. (XLSX)

S1 Table Detailed composition of Average, Healthy and Vegetarian diets. (XLSX)

S2 Table Modifications in the composition of animal rations (as percentage-point decrease or increase in mass) and animal-production changes per animal type for life cycle inventories of production systems providing higher omega-3 levels, compared to life cycle inventories of AGRIBALYSE 1.1 production systems. ADG: Average Daily weight Gain.
(XLSX)

S3 Table Eicosapentaenoic Acid (EPA) and Docosahexaenoic Acid (DHA) values for standard and BBC animal foods provided by the Bleu-Blanc-Coeur association. (XLSX)

S4 Table Environmental impacts of eight diets. (XLSX)

S5 Table Life cycle impact assessment results for food items. (XLSX)
